# Non-immune Prophylaxis Against COVID-19 by Targeting Tolerance for Angiotensin II-Triggered SARS-CoV-2 Pathogenesis

**DOI:** 10.3389/fmed.2021.776903

**Published:** 2022-01-12

**Authors:** Michael V. Dubina

**Affiliations:** ^1^State Research Institute of Highly Pure Biopreparations, St. Petersburg, Russia; ^2^Russian Academy of Sciences, Moscow, Russia

**Keywords:** COVID-19, SARS-CoV-2, angiotensin II, pathophysiology, prophylaxis, tolerance of infection

It is a joke in Britain to say that the War Office is always preparing for the last war. But this is probably true of other departments and of other countries …

Winston S. Churchill (1)

## Introduction

As of December 12, 2021, 5.3 million deaths had been reported worldwide due to the coronavirus disease COVID-19, which is caused by severe acute respiratory syndrome coronavirus (SARS-CoV)-2 (2). The effects of infection with this virus range from asymptomatic infection to “ground-glass” pneumonia with lower airway collapse, which can result in life-threatening complications (3). Previous studies of SARS-CoV infections have shaped current medical opinion on the important features of COVID-19 pathogenesis, including viral entry, replication, and migration down the respiratory tract, resulting in airway epithelium damage, diverse immune responses, inflammation, hypoxia, and acute respiratory distress syndrome (4).

For other SARS-CoVs, the receptor for SARS-CoV-2 on the host cell surface is angiotensin-converting enzyme 2 (ACE2) (5); this enzyme reduces the local and circulating levels of angiotensin II (Ang II), which is produced by angiotensin-converting enzyme (ACE) and is a major part of the renin–angiotensin–aldosterone system and is involved in blood pressure regulation (6).

This article discusses the involvement of ACE2, Ang II, and SARS-CoV-2 in the pathogenesis of lung damage in COVID-19. Based on these considerations, it proposes prophylactic measures that could maintain the lungs' capacity for dilation, redox, and metabolic functions to protect against high-risk SARS-CoV-2 infection.

## SARS-CoV-2 Pathophysiology and ANG II

Early studies of SARS-CoV found that its receptor, ACE2, has a protective role in acute lung failure *in vivo*, as opposed to ACE (7). Moreover, experimental SARS-CoV infections of wild-type mice resulted in reduced ACE2 protein expression in the lungs, and the decreased *Ace2* gene expression in knockout mice resulted in low infectivity of SARS-CoV (8). Similar to other virus-receptor interactions, when SARS-CoVs interact with ACE2 to gain entry into cells, they are endocytosed with the virus, and this loss of ACE2 from the cell surface results in increased levels of local and circulating Ang II (9). Since ACE2 is found not only in nasal and alveolar epithelial cells but also in the smooth muscle and endothelial cells of the airways (10, 11), the increased local production of Ang II may have a profound constrictive effect on the microvasculature and distant bronchioles. In particular, Ang II targets smooth muscle cells in the vascular wall, causing internalization and degradation of membrane potassium channels (12) and inducing vasoconstriction by activating angiotensin II receptor type 1 (13).

SARS-CoV-2 has a greater binding affinity for ACE2 than other SARS-CoVs (14, 15). Indeed, SARS-CoV-2 can severely inhibit ACE2 activity and reduce Ang II consumption, resulting in abnormally high levels of Ang II in the airways (16–18), impairing metabolic homeostasis in smooth muscle cells (19), and inducing pulmonary vasoconstriction (20). Such Ang II-induced pulmonary vasoconstriction also occurs in hypoxia and can be restored by concomitant upregulation of ACE2 (21). Transcriptome analyses after Ang II infusion *in vivo* have revealed upregulation of genes involved in metabolism, whereas genes that are protective against oxidative stress were downregulated (22). Thus, vaso- and bronchoconstriction resulting from transient virus-induced Ang II elevation in distal airways might precede epithelial damage and immune responses in the initial phase of the infection. The virus-induced increase in Ang II in infected lungs is probably a key local event that promotes the contraction of arterioles and cartilage-free terminal bronchioles, resulting in lower airway collapse, pulmonary hypoxia, microcirculation reduction, impaired airway cell metabolism, and diffuse alveolar damage.

Ang II triggers a vicious cycle of pathogenesis in the lungs, as vasoconstriction decreases its subsequent elimination. In particular, SARS-CoV-2-induced downregulation of ACE2 impairs Ang II clearance and may aggravate lung damage (23). Of note, plasma levels of ACE2 and Ang II do not correlate with the clinical outcomes in patients with COVID-19 (24); thus, the concentration of Ang II in the circulation is not a reliable indicator of its local concentration in the lower airways where Ang II-induced vasoconstriction may reduce the pulmonary microcirculation.

Distinctive from immunological resistance to infection, another component of the host defense response against pathogens is tolerance, which depends on the ability of the body to regulate the production, repair and avoidance of the damage accumulated during an infection (25). In line with this concept, the initiation and severity of lung pathology due to a rapid virus-mediated increase in local Ang II levels may depend on the innate capacity of the lungs to mitigate vasoconstriction by increasing dilation as well as increasing redox and metabolic functions. Infected individuals with poor pulmonary, cardiovascular, and metabolic conditions may thus suffer from greater vascular resistance in the airway periphery, thereby reducing the ventilation–perfusion properties of the lungs and causing severe COVID-19 complications. This might explain the lower tolerance with the greater severity and mortality observed in COVID-19 patients with comorbidities (26).

## Uncertain Efficacy of Current COVID-19 Therapeutics and Prevention

The first strategies developed by the World Health Organization and national health care authorities included oxygen support and antibiotic treatments for patients and public health measures to reduce community transmission (27). The numerous treatments being studied include off-label antiviral drugs and drugs that combat hypoxemia and coagulation disorders, in addition to immunomodulatory and anti-inflammatory therapies using convalescent plasma, glucocorticoids, and anticytokines (28, 29). Studies using ACE inhibitors or Ang II type 1 receptor blockers and/or delivering recombinant ACE2 to the lungs have been initiated in hospitalized patients with COVID-19 (30). Moreover, there has been an unprecedented international effort by private and public institutions to develop vaccines against SARS-CoV-2 (31). Recent systemic reviews have reported that adenovirus vectors and mRNA vaccines are 65–95% effective at preventing SARS-CoV-2 infection after full vaccination (32, 33).

On October 15, 2020, the world's largest randomized controlled trial on COVID-19 therapeutics found that the previously recommended remdesivir, hydroxychloroquine, lopinavir/ritonavir, and interferon regimens had little or no effect on overall mortality, initiation of ventilation, or improved prognosis of COVID-19 among hospitalized patients (34). The effects of convalescent plasma, glucocorticoids, antibiotics, anticoagulants, and other previously repurposed drugs were also insignificant (35, 36). The use of ACE inhibitors or Ang II type 1 receptor blockers had no clear association with COVID-19 incidence and all-cause mortality; they may be protective for patients with hypertension (37, 38). Social distancing and seasonality still have a greater influence on infection rates than vaccination, and new virus variants threaten renewed outbreaks. The UK, Germany, and Canada, for example, are facing a new wave of SARS-CoV-2 infection despite having some of the highest vaccination rates and strict ongoing lockdown restrictions (39). To date, no agents have proven effective at preventing SARS-CoV-2 infection (40, 41).

## A Novel Strategy for COVID-19 Management

In a pilot study, 99 health care workers at a COVID-19 hospital were treated prophylactically with an aerosol containing glutathione, potassium, and inosine at low doses for 14 days to promote lung function (42). The rationale for this combination of drugs was that glutathione decreases smooth muscle contraction (43) and induces bronchodilation by increasing membrane hyperpolarization via potassium channels (44); extracellular potassium increases blood flow in the lungs, thus improving vasodilation (45); and inosine boosts adenosine triphosphate generation, thus protecting cells from hypoxia (46). The study found 78% efficacy (i.e., relative risk reduction in the test group compared to a control group) of this pathogenesis-based prophylaxis against SARS-CoV-2 infection. Only five participants reported mild and transient adverse effects.

The efficacy and safety of these low-dose compounds when delivered to the lung as an aerosol (47, 48) suggests the potential of this prophylactic strategy to prevent SARS-CoV-2 infection and to fight against COVID-19 by directly promoting lung capacity for dilation, as well as redox and metabolic functions. Amelioration of the initial virus-induced, Ang II-triggered pathology in the small airways by this or similar inhaled dilatation and antihypoxic therapies might appreciably prevent the occurrence and/or severity of the disease.

Furthermore, numerous available well-tolerated medications, such as β2–adrenergic agonists, phosphodiesterase III inhibitors, and superoxide dismutase, might be repurposed to better manage the disease. The potential of this tolerance-targeted prophylaxis to defend against the disease and to improve treatment would be unaffected by mutations in the virus, as the proposed agents are not antiviral *per se* but are intended to break the initial vicious cycle of COVID-19 pathogenesis triggered by SARS-CoV-2 entry and replication.

## Author Contributions

The author confirms being the sole contributor of this work and has approved it for publication.

## Conflict of Interest

The author declares that the research was conducted in the absence of any commercial or financial relationships that could be construed as a potential conflict of interest.

## Publisher's Note

All claims expressed in this article are solely those of the authors and do not necessarily represent those of their affiliated organizations, or those of the publisher, the editors and the reviewers. Any product that may be evaluated in this article, or claim that may be made by its manufacturer, is not guaranteed or endorsed by the publisher.
